# Cathode–Electrolyte Interface Modification by Binder Engineering for High‐Performance Aqueous Zinc‐Ion Batteries

**DOI:** 10.1002/advs.202205084

**Published:** 2022-12-16

**Authors:** Haobo Dong, Ruirui Liu, Xueying Hu, Fangjia Zhao, Liqun Kang, Longxiang Liu, Jianwei Li, Yeshu Tan, Yongquan Zhou, Dan J.L. Brett, Guanjie He, Ivan P. Parkin

**Affiliations:** ^1^ Christopher Ingold Laboratory Department of Chemistry University College London 20 Gordon Street London WC1H 0AJ UK; ^2^ Electrochemical Innovation Lab Department of Chemical Engineering University College London 20 Gordon Street London WC1E 7JE UK; ^3^ Key Laboratory of Comprehensive and Highly Efficient Util Laboratory of Salt Lake Resources Chemistry of Qinghai Province Chinese Academy of Sciences Xining Qinghai 810008 China; ^4^ Materials and Catalysis Laboratory Department of Chemical Engineering University College London London WC1E 7JE UK

**Keywords:** in situ formation, interface engineering, water‐soluble binder, zinc‐ion batteries

## Abstract

A stable cathode–electrolyte interface (CEI) is crucial for aqueous zinc‐ion batteries (AZIBs), but it is less investigated. Commercial binder poly(vinylidene fluoride) (PVDF) is widely used without scrutinizing its suitability and cathode‐electrolyte interface (CEI) in AZIBs. A water‐soluble binder is developed that facilitated the in situ formation of a CEI protecting layer tuning the interfacial morphology. By combining a polysaccharide sodium alginate (SA) with a hydrophobic polytetrafluoroethylene (PTFE), the surface morphology, and charge storage kinetics can be confined from diffusion‐dominated to capacitance‐controlled processes. The underpinning mechanism investigates experimentally in both kinetic and thermodynamic perspectives demonstrate that the COO^−^ from SA acts as an anionic polyelectrolyte facilitating the adsorption of Zn^2+^; meanwhile fluoride atoms on PTFE backbone provide hydrophobicity to break desolvation penalty. The hybrid binder is beneficial in providing a higher areal flux of Zn^2+^ at the CEI, where the Zn‐Birnessite MnO_2_ battery with the hybrid binder exhibits an average specific capacity 45.6% higher than that with conventional PVDF binders; moreover, a reduced interface activation energy attained fosters a superior rate capability and a capacity retention of 99.1% in 1000 cycles. The hybrid binder also reduces the cost compared to the PVDF/NMP, which is a universal strategy to modify interface morphology.

## Graphical abstract

Intensive efforts are applied to cathode, anode, and electrolyte, while it still lacks a comprehensive understanding of the interface mechanisms. Herein, a universal strategy is proposed for stabilizing the cathode directly from interface modification by the binder interface engineering. After introducing anionic polyelectrolyte in the binder, the battery interface mechanism can be tuned between diffusion control and capacitive dominance.

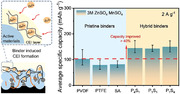

## 1 Introduction

**Scheme 1:**
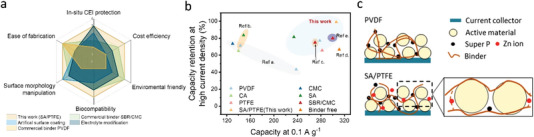
a) Radar diagram of the comparison of current strategies to stabilize the battery performance of AZIBs; b) summary of binder developments for AZIBs, where the highlighted areas are from the literature. (Ref a. [27], Ref b. [28], Ref c. [29], Ref d. [30], Ref e. [31]); c) schematic diagrams comparing the conventional binder PVDF and the SA/PTFE hybrid binder in this work.

Because of limited raw materials and combustible organic electrolytes, lithium‐ion batteries (LIBs) have encountered drastic price inflation and safety issues, which have raised serious concerns when utilizing LIBs for large‐scale applications.^[^ ^1^ ^]^ Rechargeable aqueous zinc‐ion batteries (AZIBs) have been regarded as alternative candidates, especially for grid/off‐grid applications due to their advantages in economic efficiency,^[^ ^2^ ^]^ intrinsic safety,^[^ ^3^ ^]^ and high theoretical volumetric energy density (5855 mAh cm^−3^) of Zn metal anodes.^[^ ^3^ ^]^ Compared with vanadium‐based oxides and Prussian blue analogues, Zn‐MnO_2_ batteries have attracted much attention due to their abundant sources and minimal environmental impact.^[^ ^4^, ^5^ ^]^ Owing to the large ionic radius of hydrated Zn^2+^ solvent sheaths (4.7 Å),^[^ ^6^ ^]^ the strong electrostatic interactions between cathode structures and inserting charge carriers result in sluggish diffusion kinetics and poor rate performance.^[^ ^7^ ^]^ Extensive efforts have been evaluated to solve the desolvation at the electrode–electrolyte interface,^[^ ^8^, ^9^ ^]^ such as design of pillar cathode materials,^[^ ^10^ ^]^ electrolyte modification,^[^ ^11^ ^]^ and anode treatment.^[^ ^12^ ^]^ As summarized in **Scheme** **1**a, strategies consist of electrolyte modifications, such as water‐in‐salt solution and anode surface coatings, are reported to stabilize the cathodes and anodes. However, such strategies, either highly expensive or acquiring additional fabrication, are not beneficial for commercialization. Thus, a universal and cost efficient interface confinement is essential to stabilizing active cathode materials.^[^ ^8^, ^13^ ^]^ The binder acts as a dispersion agent and adheres active materials to the current collector and is a critical component at the electrode–electrolyte interface. Compared to the electrolyte and electrode modification, the binder is the most cost‐effective component, with only 1–2% of the material cost breakdown,^[^ ^14^ ^]^ which could be optimized without additional operational cost in the production process. However, conventional binder polyvinylidene fluoride (PVDF) only offers a basic function of adhering cathode materials and has no extra benefits, such as stabilizing active materials. Most research conducted to date has emphasized active materials while less attention being devoted to the binder recipes when investigating interfacial modification. Herein, to provide a universal strategy, a dual‐functional water‐based binder has been developed for AZIBs. An in situ formed cathode–electrolyte interface (CEI) protecting layer is initiated during the galvanostatic cycling process, thus endowing a long‐duration stability. In the meantime, the binder also modifies the interface charge‐transfer mechanism to deliver high‐rate capabilities.

Typically, a cathode was prepared by mixing active materials with conductive carbon and a polymeric binder in a solvent, followed by casting the slurry onto current collectors.^[^ ^15^ ^]^ In general, PVDF is utilized as a binder because of its good thermal stability and adhesion capability. However, this was originally designed for batteries with organic electrolytes, especially LIBs, whereas there is limited investigation into aqueous batteries. *N*‐methyl‐2‐pyrrolidone (NMP) is required as a solvent to form the PVDF slurry, which is toxic, causing central nervous system dysfunction and depression, and is also environmentally unfriendly and expensive. As stated in Scheme 1b, there is a lack of systematic study of binders, such as the effect of the wettability for binder in electrochemical performances for AZIBs or even other aqueous multivalent metal‐ion batteries. Yushin et al.^[^ ^16^ ^]^ have attributed the substantial volume variation during charge carrier insertion with the importance of the binder critically related to battery stability and irreversible capacity loss. The weak Van der Waals interactions between active materials and current collectors consequently induce a fast capacity decay under significant volume changes, especially for sulfur and silicon electrodes.^[^ ^17^, ^18^, ^19^ ^]^ By strengthening the adhesion properties, water‐soluble polymer binders, either natural, or synthesized, have been confirmed to provide strengthening chemical/physical interactions and eventually improve electrode stability,^[^ ^17^, ^20^, ^21^ ^]^ especially for polysaccharides.^[^ ^22^, ^23^, ^24^ ^]^ Hence, sodium alginate (SA), a typical polysaccharide, was chosen as one of the polymer binder frameworks. Due to the coordination affinity for divalent metal ions from the SA backbone,^[^ ^25^, ^26^ ^]^ there is a simultaneous ionic conductive gel formation between carboxyl moieties and Zn^2+^ ions during the cycling process forming the helical structure, thus protecting the cathode from structural collapse (Scheme 1c). As reported by Yushin, because of the ionic bonding, SA also exhibits ≈6.7× higher stiffness than the commercial binder, PVDF.^[^ ^16^ ^]^ Therefore, alginate not only strengthens the adhesion but exhibits the capability to accommodate cathode volume expansion, thus avoiding structural collapse. The polytetrafluoroethylene (PTFE) has also been applied as the binder to manipulate an appropriate surface morphology, where the hydrophobic interaction of ‐CF in PTFE backbones facilitates the desolvation behavior of Zn^2+^. In this work, investigations were conducted at different mass ratios between SA and PTFE. Compared to PVDF/NMP, AZIBs with a newly developed binder exhibits a ≈6.1% higher specific capacity at 0.1 A g^−1^, and especially at 2 A g^−1^, the capacity is 47.5% greater than the one for AZIBs with PVDF. An optimal binder composition was determined with a weight ratio of PTFE:SA = 1:4 noted as P_1_S_4_. Owing to the in situ protective layer formation on cathodes, the bifunctional binder exhibits superior capacity retention of 99.1% over 1000 cycles at a current density of 2 A g^−1^. The energy storage mechanism of the hybrid binder was elucidated via both kinetic and thermodynamic studies, where Zn^2+^ diffusion coefficient and surface activation energy were evaluated respectively. With different composition of SA and PTFE, the interfacial mechanism was modified from diffusion‐controlled to capacitance‐dominated processes. Compared to the previous work (Scheme 1b), it delivers the best‐in‐class performance. This interfacial treatment strategy can be also extended to other aqueous multivalent metal‐ion batteries.

## 2 Results and Discussion

**Figure 1:**
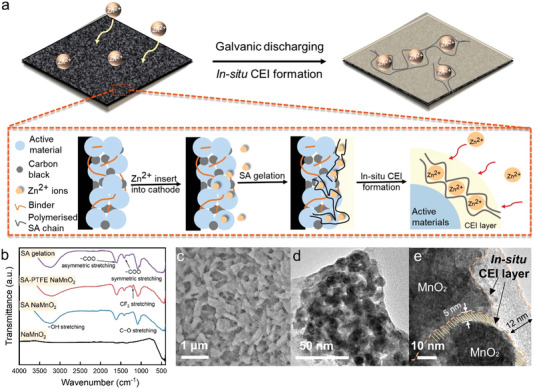
In situ formation of CEI layer. a) schematic diagram of the in situ formation of the protective CEI layer; b) FTIR spectra of the mixture of NaMnO_2_ cathode materials and the composite binder after immersing into the electrolyte; c) SEM image of the cathode mixture after cycling (30 cycles at 0.1A g^−1^); d,e) TEM images of the cathode mixture after cycling (30 cycles at 0.1A g^−1^) where the light grey and shaded region are the CEI hydrogel layer.

The hybrid binders were prepared by mixing SA and PTFE in an aqueous solution. Different compositions of PTFE and SA were prepared and noted as P_4_S_1_, P_1_S_1_, P_1_S_4,_ P_1_S_6_, and P_1_S_8_ which correspond to the weight ratios of PTFE:SA of 4:1, 1:1, 1:4, 1:6, and 1:8 respectively. As for comparison, conventional binder PVDF was also prepared by dissolving the PVDF powder into an NMP solution with a concentration of 5 wt% (5 mg in 100 µL). Na^+^ pre‐intercalated MnO_2_ was applied as the active material synthesized under a facile co‐precipitation method as reported in our previous work.^[^ ^32^ ^]^ Owing to the merits, the intrinsic affinity of SA to divalent metal ions, the linear polysaccharide^[^ ^33^ ^]^ is crosslinked by Zn^2+^ and Mn^2+^ ions in the aqueous electrolyte, offering a superior adhesion between active materials and the current collector. As an analogue to the hydrogel electrolyte, the crosslinked binder could also be regarded as a thin layer of SA‐based hydrogel electrolyte formed at the CEI. As indicated in the schematic diagram (**Figure** **1**a), during galvanostatic discharge, intercalated Zn^2+^ ions in the cathode initiate in situ gelation of hydrogel propagating from the CEI to the particulate interval of active materials. Material characterizations by Fourier transform infrared (FTIR) spectra, scanning electron microscopy (SEM) and transmission electron microscope TEM were performed to observe the gelation process. To deliver clear patterns and microscopic images without the influence of conductive carbon and carbon paper substrates on the cathode, the cathode was prepared on the titanium foil substrates without Super P powders for these characterizations.

FTIR spectra (Figure 1b) correspond with an in situ gelation mechanism that was induced simultaneously when the cathodes with binders were immersed into the electrolyte. The characteristic transmittance of the active materials, sodium alginate, and sodium alginate after gelation was determined as the baseline. Compared to the pristine cathode, the presence of characteristic absorption bands at 1612 and 1420 cm^−1^, also shown in the top spectrum, indicates the divalent crosslinking with carboxylic groups at the backbone, which belongs to asymmetric and symmetric —COO stretching. The characteristic peaks at 1082 and 1028 cm^−1^ are assigned to C—O stretching in CH—OH and C—O—C structures, respectively. The broader band from 3000–3600 cm^−1^ is ascribed to the —OH stretching referring to absorbed water molecules. Moreover, C—H deformation was detected at 1298 cm^−1^. After complexing PTFE into the binder, characteristic peaks at 1222 and 1155 cm^−1^ were observed, which were attributed to asymmetrical and symmetrical —CF_2_ stretching, respectively.^[^ ^34^, ^35^, ^36^ ^]^ SEM images also confirm the in situ CEI formation. As shown in Figure S1a, Supporting Information, the cathode with the hybrid binder PTFE‐SA before cycling has no specific morphology but shows bead‐like or lump‐like particles, which resulted from the unlinked alginate. While after cycling, clear porous structures were attained, as shown in Figure 1c, which are assigned to the gelation of SA. Compared to the one with PVDF binder, it only demonstrates a lump‐like morphology of the cathode as shown in Figure S2b, Supporting Information. As confirmed by TEM images, Na‐Birnessite MnO_2_ particles were wrapped by the SA layer. As displayed in low‐magnification TEM images, the dark areas belong to the active material, while the light grey borders are crosslinked sodium alginate covering the active material. Under high magnification (Figure 1e), the highlighted region could be assigned to the cathode electrolyte interface. Detailed images from a scanning transmission electron microscope (STEM) further verified the morphology. As displayed in Figure S1b,c, Supporting Information, an amorphous polymer layer was attained for the cathode after cycling. CEI layer located at the edge surrounds the active material with the thickness of 5.4 nm after 30 cycles. As shown in the energy dispersive spectroscopy (EDS) mapping images (Figure S3, Supporting Information), the uniformly distributed Zn and Mn indicate the homogeneity of the cathode with the hybrid binder after gelation. The porous structure indicates the presence of hydrogel protecting layer for the hybrid binder in the aqueous electrolyte, in the meantime, EDS mapping also reveal the uniform of the protecting CEI layer. As state by Wu,^[^ ^37^, ^38^, ^39^ ^]^ a smooth and uniform surface is often ideal for the improvement of cycle life and capacity, therefore the in situ CEI layer is essential to enhance the battery performance. In terms of the XRD spectrum, the gelation of the hybrid binder also results in a more amorphous structure compared to pristine Na‐Birnessite MnO_2_ (Figure S4, Supporting Information). While as for cathodes with the conventional binder, there is no protecting CEI layer except from the formation of flower‐like Zn_4_SO_4_(OH)_6_·5H_2_O (Figure S14, Supporting Information).

**Figure 2:**
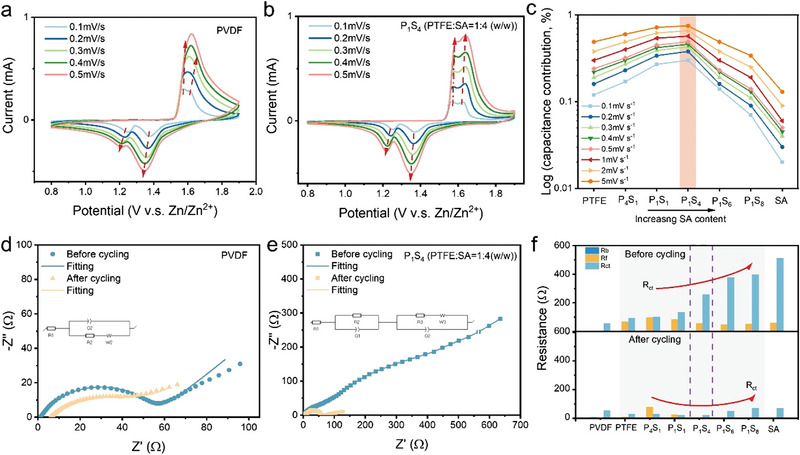
CV and EIS performances. a) CV of ZIBs with pristine PVDF binder; b) CV of ZIBs with the hybrid binder PTFE:SA = 1:4 at scan rates of 0.1, 0.2, 0.3, 0.4, and 0.5 mV s^−1^; c) capacitance contribution for ZIBs by using hybrid binders with different compositions (*y*‐axis is the logarithm of the scan rate); d) EIS of the pristine PVDF binder; e) EIS of the hybrid binder with PTFE:SA = 1:4; f) impedance analysis of ZIBs using different binders, where internal resistance (*R*_b_), interface resistance (*R*_f_), and charge‐transfer resistance (*R*_ct_) are compared.

Multiple electrochemical performances including CV, EIS, and galvanostatic charge/discharge were investigated for Zn//Na_0.65_Mn_2_O_4_∙1.31H_2_O full cells in the 3 m ZnSO_4_ and 0.2 m MnSO_4_ electrolyte. CV profiles were investigated for ZIBs using hybrid binders with different compositions of PTFE, SA, P_4_S_1_, P_1_S_1_, P_1_S_4_, P_1_S_6_, and P_1_S_8_. As shown in **Figure** **2**b, the hybrid binder exhibits consistent two redox couples similar as the one with PVDF binders, where at a low scan rate of 0.1 mV s^−1^, two reduction peaks are 1.37 and 1.25 V, and oxidation peaks are located at 1.56 and 1.63 V. The two‐step electrochemical processes are ascribed to the H^+^ and Zn^2+^ intercalation during discharge processes at 1.37 and 1.25 V respectively, whereas the corresponding oxidation peaks are associated with Zn^2+^ de‐intercalation and the reduction of Mn^4+^ to Mn^3+^. Conventionally, with the increase of scan rates, two redox peaks merge into a broader peak (Figure 2a), while as a comparison between the hybrid binder SA/PTFE and PVDF, the hybrid binder maintains a clearly separated redox couple even at a high scan rate of 0.5 mV s^−1^. The formation of a CEI layer facilitated well‐maintained CV curves and was essential to deliver high‐capacity retention by improving the ion diffusion.

According to Dunn's power‐law relation,^[^ ^40^ ^]^ the energy kinetic mechanism was quantified by a diffusion model with diffusion‐controlled and surface‐controlled charge storage processes.^[^ ^41^ ^]^ Hence the voltammetry can be deconvoluted into two current responses, a current response obeying the diffusion‐controlled mechanism for ion intercalation/deintercalation, and a capacitive process as shown by the following Equation (1), where *k*_1_ *v* is ascribed to the capacitive current and *k*_2_ *v* ^1/2^ is ascribed to the diffusion‐limited current. Furthermore, Equations (2) and (3) derived also provide a parameter to realize the kinetic mechanism for the cathode related to the peak current response, where there is a diffusion control from the intercalation mechanism if 0.5 < *b* < 0.7, otherwise it is a surface controlled process if *b* is close to 1.^[^ ^42^ ^]^

(1) $$i=k_{1}v+k_{2}v^{1/2}$$

(2) $$i_{peak}=av^{b}$$

(3) $$\mathrm{hence},\log\left(i_{peak}\right)=\log\left(a\right)+b\cdot \log\left(v\right)$$

As shown in Figure S5, Supporting Information, ZIBs with commercial PVDF binder exhibit a diffusion‐controlled behavior where *b* is less than 0.6. Similarly, as for ZIBs with pristine PTFE and sodium alginate, electrolytic ion intercalation/deintercalation dominates in the diffusion model, especially for sodium alginate, magnitudes of *b* are ≈0.5 for all the calculated peaks. However, complexing PTFE with SA, ZIBs with hybrid binders even exhibit capacitive kinetics, where the average *b* values are 0.653, 0.701, and 0.768 for P_4_S_1_, P_1_S_1_, and P_1_S_4_ respectively, indicating that *i*_peak_ departs drastically from the capacitive control in the relation of *i*_peak_ versus *v* ^1^. With increasing sodium alginate contents, there is an increasing domination of capacitive kinetics deduced from the increased *b* values; however, further increase in alginate content after mass ratio 1:4 resulting a decrease in capacitive contribution to diffusion control, for which the average *b* values are 0.749 and 0.554 for P_1_S_6_ and P_1_S_8_ respectively. Hence, the interface can be modelled as Nernst diffusion layer model as reported by Bergel,^[^ ^42^ ^]^ for which the influence to the diffusion mechanism from the concentration gradient of the charge carrier at the interface cannot be neglected. The quantitative contribution derived from Dunn's equation also elucidated an increased capacitive contribution. Instinctually, as the sweep rate increases, surface capacitance dominates the kinetic process where the contribution of diffusion‐controlled behavior is reduced. Following with the logarithmic conversion shown in Figure 2c, a summary of the contribution ratio of capacitance for ZIBs with hybrid binders demonstrates that with the increasing mass ratio of SA to PTFE, increased capacitive contributions reach to an optimal magnitude at P_1_S_4_ followed by a decreasing tendency with a further increasing content of alginate. As for the ZIB with P_1_S_4_ (PTFE:SA = 1:4 ratio in weight), it possesses a capacitive contribution ≈18% greater than the one with PTFE at 0.1 mV s^−1^, while at a high sweep rate of 5 mV s^−1^, this difference raises to 26%. Figure S6, Supporting Information clearly shows this phenomenon, for which the enclosed pink regions increase along with the compositional variation. In contrast to PVDF, a capacitive analysis of ZIBs with hybrid binders reveals the kinetics is dominated by surface‐controlled processes. Under a further increased composition of SA, diffusion‐control is dominate the kinetics which is similar to the one for pristine SA.

Nyquist plots presented in Figures 2d,e unveil the variation of impedances before and after 30 cycles for ZIBs with hybrid binders and PVDF. As for ZIBs with PVDF, the single semi‐circle obtained reflects an equivalent circuit without surface resistance. Compared to impedances before cycling, there was a typical increase of bulk resistance *R_b_* from 1.16 to 4.49 Ω, which was assigned to the ageing issue in the aqueous electrolyte,^[^ ^43^ ^]^ whereas a slight decrease attained of *R*_ct_ from 55.19 to 53.92 Ω indicates a stable charge transfer kinetics resulting from the commercial binder. Nevertheless, as for ZIBs with hybrid binders, for example P_1_S_4_, an obvious semi‐circle related to the interface (*R*_f_) was attained at the high frequency in addition to the charge transfer impedance addressed from the medium frequency to low frequency (Figure 2e). After cycling, it is noteworthy that a broader semi‐circle for the hybrid binder is absent, resulting in a reduced *R*_ct_ from 258.09 to 21.31 Ω. Meanwhile, *R*_f_ also decreases from 57.1 to 5.9 Ω. Before cycling, the un‐crosslinked SA results in a high charge transfer resistance for Zn^2+^ ions to intercalate into layered cathode structures, where Zn^2+^ ions are affiliated with linear SA initiating the gelation process rather than direct cathode intercalation. Before cycling, a less conductive cathode electrolyte interface also causes the presence of interfacial impedance. However, during cycling, the in situ gelation process of the alginate binder is activated by the Zn^2+^ insertion/extraction process and eventually results in a superior Zn^2+^ conductive cathode‐electrolyte interface layer after cycling, hence delivering a reduced *R*_f_ and *R*_ct_. Figure 2f shows the resistance variation for different compositions of hybrid binders listed in Table S1, Supporting Information. Owing to the impedance induced by the gelation process, *R*_ct_ raised with an increase of sodium alginate content before cycling. While after cycling, *R*_ct_ values attained for hybrid binders were <30 Ω, reaching the value similar to the one for pristine PTFE (29.75 Ω). However, further increasing the SA ratio above 1:4 resulted in a three‐fold increase of *R*_ct_ where there was an increasing resistance after the optimum magnitude for P_1_S_4_. In terms of *R*_f_, because of the formation of the conductive layer, magnitudes of the interfacial impedance after cycling decrease with the increasing amount of SA reaching a similar value as SA (5.9 Ω). With the application of SA, the ionic conductive binder reduces the barrier for the interfacial impedance between active materials. Interestingly, the hybrid binder exhibits both merits of SA and PTFE in *R*_f_ and *R*_ct_ without retaining their respective drawbacks, providing a “win–win” performance. The reason behind this was further evaluated by Arrhenius analysis and the DFT simulation as illustrated in the Supporting Information, where a desolvation mechanism at the cathode‐electrolyte interface will be discussed thereinafter.

**Figure 3:**
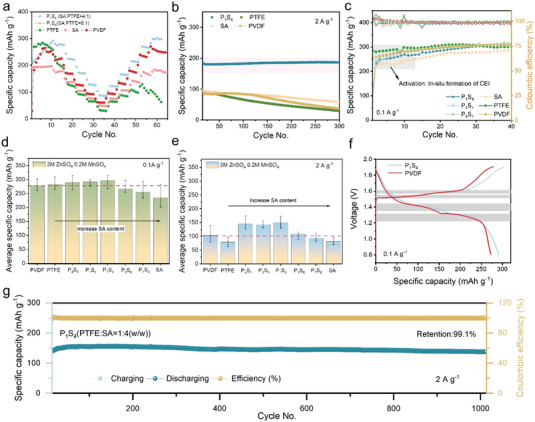
Galvanostatic charging/discharging performance. a) Rate performance under current densities of 0.1, 0.2, 0.5, 1, 2, and 5 A g^−1^; b) long‐term cycling performance at 2 A g^−1^; c) Cycling activation for in situ formation of CEI; d) average specific capacity of AZIBs with different binders at 0.1 A g^−1^; e) average specific capacity of ZIBs with different binders at 2 A g^−1^; f) specific capacity profiles of ZIBs with PVDF and P_1_S_4_ at 0.1 A g^−1^; g) long‐duration stability performance of AZIBs with the P_1_S_4_ binder at the current density of 2 A g^−1^.

Galvanostatic charging/discharging (GCD) performances were investigated in both rate and long‐term stability tests. To ensure the accuracy and repeatability of the experiments, cathodes were prepared and evaluated in different coin cells. All GCD experiments were examined at least five times, and in the meantime, the average magnitudes of specific capacity were used for comparison to minimize the influence of extreme values. As shown in **Figure** **3**a, rate charging/discharging tests were performed under different current densities for ZIBs with both hybrid and commercial binders. Compared to the one with PVDF, ZIBs with hybrid binders exhibit a better rate performance as addressed in the highlighted region. For example, ZIBs with the composition PTFE: SA = 1:4 (P_1_S_4_) binder exhibits 10% greater specific capacities of 291, 276, 225, 172, 145, and 101 mAh g^−1^ at corresponding current densities compared to the one with PVDF binder of 278, 264, 231, 178 106, and 77 mAh g^−1^ under the same current densities of 0.1, 0.2, 0.5, 1, 2, and 5 A g^−1^. ZIBs with pristine PTFE could reach a high capacity of 285 mAh g^−1^ at a low current of 0.1 A g^−1^, while its inadequate rate performance results in the lowest specific capacity of 31 mAh g^−1^ at 5 A g^−1^. AZIBs with alginate demonstrate a lower capacity variation in the GCD rate performance from 48 to 197 mAh g^−1^. Comparisons for the hybrid binders with different compositions shown in Figure S9a, Supporting Information further reveal the differences in rate performance. As indicated in Figure 3d,e, average specific capacities were calculated for comparison. At a low current density of 0.1 A g^−1^, a longer diffusion time of charge carriers results in similar capacities delivered except pristine sodium alginate, which is 15.8% less than PVDF. Regarding other binders, there is only a 5.9% difference between the hybrid binder (P_1_S_4_) and commercial PVDF. Whereas under a high current density of 2 A g^−1^, as shown in the bar chart (Figure 3e), specific capacities attained for ZIBs with hybrid binders are ≈40% greater than the one with the PVDF binder. Especially, as for the ones with P_1_S_4_, these ZIBs exhibit a 45.6% difference in the contrast to the one with the PVDF, reaching an average capacity of 149.8 mAh g^−1^. Increasing the SA further reduces the obtained capacity to the capacity of the pristine SA. Considering the diffusion‐capacitance process, the higher average capacity obtained at the high current density can be illustrated by the capacitance‐controlled kinetics. This is attributed to the increasing capacitive response of the hybrid binder with increasing sodium alginate content, superior pseudocapacitances stored at the cathode electrolyte interface were expected under a high current density, hence a relatively higher capacity than ZIBs with pristine PTFE and sodium alginate binders. While at a low current density, due to the domination of diffusion‐controlled kinetics, there is limited capacitance effects. With a specific emphasis on the GCD profile at low current densities (0.1A g^−1^), a CEI activation process was notified in the first 40 cycles as addressed in Figure 3c. In the shaded region, ZIBs with PVDF and PTFE binders exhibit a shorter activation process with 15 cycles to reach a stable maximum capacity, whereby a mildly raising slope was attained for sodium alginate throughout the 40 cycles. Due to the gelation of linear SA structure on the cathode, the formation of in situ CEI requires a longer Zn^2+^ diffusion time from CEI to the active material, hence contributing to a longer activation period. Consistent with this observation in impedance variation, a reduced *R*_ct_ of ZIBs with SA after cycling also verified the activation progress of the in situ CEI formation. However, because of the mono‐function of adhesion for PVDF and PTFE, there is only slight activation of inactive materials, hence contributing to a shorter activation period. Consequently, the activation cycles of ZIBs with hybrid binders were between 15 and 40 cycles, and a shorter activation time was accompanied by a higher PTFE composition. Increasing the SA mass ratio in the binders, the duration required for activation is about 15, 20, and 25 cycles for P_4_S_1_, P_1_S_1_, and P_1_S_4_, respectively, indicating a general correlation between the activation process and SA composition. However, as indicated in Figure 3f, the reaction plateaus have not been influenced during the CEI formation for the ZIBs with hybrid binder compared to PVDF. Both profiles exhibit typical two‐stage voltage plateaus for which two plateaus at 1.45 and 1.3 V at the discharging process, and two plateaus at 1.5 and 1.6 V. Typically, H^+^ and Zn^2+^ ions co‐insertion dominate the energy storage mechanism in zinc‐ion batteries.^[^ ^8^, ^44^, ^45^ ^]^ Considering the hydrated radius between H^+^ and Zn^2+^, the first plateau during discharging (1.45 V) is assigned to the intercalation of H^+^ ions, while Zn^2+^ intercalations happen at the second plateau (1.3 V). In the meantime, the valence states of Mn oxidized to Mn^4+^ during discharging and reduced to Mn^3+^ during charging processes.^[^ ^46^, ^47^ ^]^ The same plateaus voltage achieved for composite binder indicate the reproducibility and no influence in overpotential.

The long‐term period galvanostatic charge–discharge tests were performed at 2 A g^−1^ to assess the cathode stability, where the hybrid binder P_1_S_4_ exhibits the best capacity retention compared to the others. As shown in Figure S9b, Supporting Information, capacity retentions of ZIBs with hybrid binders increase with higher sodium alginate content, showing 81.6%, 93.1%, and 99.6% for P_4_S_1_, P_1_S_1_, and P_1_S_4_, individually, within 300 cycles, while after the optimum mass ratio P_1_S_4_, there is a decrease in capacity retention attaining 85.1% and 67.3% for P_1_S_6_ and P_1_S_8_, respectively. The activation process was performed during the initial cycles at 0.1 A g^−1^. Owing to reduced *R*_ct_ and *R*_f_ along with the reduced PTFE composition, P_1_S_4_ was chosen as a promising composition that exhibits not only excellent stability but a great rate performance. Further experiments shown in Figure 3g reveals that there is only 0.9% capacity degradation for the hybrid binder P_1_S_4_ over 1000 cycles. As stated above, it is noteworthy that with increased sodium alginate amounts in the hybrid binder, the charge storge confines the interface kinetics from diffusion to a capacitance‐controlled mechanism offering a better rate performance, and high capacity at which EDLC or pseudocapacitances resulting from an enhanced interfacial kinetics are beneficial to a competitive capacity at a high current density. Moreover, the adhesion ability evaluated by adhesive taping (see Figure S16, Supporting Information) further confirmed that the CEI layer formed in hybrid binder enhanced the adhesive bonding between active materials and the substrate compared to the one for PVDF. Consistent with the observation that polysaccharide strengthens bonding network in the electrode,^[^ ^48^, ^49^ ^]^ the formation of the designed cathode‐electrolyte interlayer protects the cathode structure from structural collapse, thus offering superior stability.

The Zn^2+^ diffusion coefficient was calculated from galvanostatic intermittent titration technique (GITT) and helped unveil the charge transfer kinetics. As seen from Figure S7, Supporting Information, Zn^2+^ diffusion coefficients for both hybrid P_1_S_4_ and PVDF are at the same magnitude of 10^−9^–10^−7^ cm^2^ s^−1^, which is consistent with the value reported by Zhi^[^ ^50^ ^]^ for Na‐intercalated MnO_2_. However, as summarized in Table S2, Supporting Information, ZIBs with P_1_S_4_ binder exhibit a 1.5× higher average diffusion coefficient compared to the one with PVDF binder in 3 m ZnSO_4_/0.2 m MnSO_4_ electrolyte. The surface activation energies were evaluated according to Arrhenius's theory based on the charge transfer resistance (*R*_ct_), further revealing the interface from a thermodynamic perspective. As shown in Figures S8a,b, Supporting Information, for both PVDF and P_1_S_4_, with rising temperature, the reduced radii of the semi‐circles reflect decreased *R*_ct_ values. Owing to the passivation phenomena resulting from the electrolyte decomposition at the electrodes, the increased *R*_b_ observed was assigned to the aggravated decomposition at higher temperatures. As indicated in Figure S8c, Supporting Information, PVDF exhibits an *E*_a_ of 26.76 kJ mol^−1^ which is of the same magnitude as the activation energy reported by Nazar et al.^[^ ^31^ ^]^ for Zn^2+^ ion inserted into vanadium‐based cathode in an aqueous electrolyte (19.47 kJ mol^−1^). However, for the hybrid binder, there is a lower barrier for Zn^2+^ ions intercalation with a lower *E*_a_ of 16.57 kJ mol^−1^. The electrolyte based on 3 m ZnSO_4_ was also evaluated for ZIBs with both binders to avoid the influence of Mn^2+^ additives, for which the same results obtained unveiling the ZIB with the hybrid binder exhibits a lower *E*_a_ (18.43 kJ mol^−1^) compared to the one with PVDF (35.57 kJ mol^−1^) (Figure S8c,f, Supporting Information). Thus, as for ZIBs with hybrid binder, the transport of hydrated Zn^2+^ ions across the CEI require a lower energy in the desolvation process compared to ZIBs with PVDF. The negatively charged alginate in the binder accelerate the Zn^2+^ intercalation by the electrostatic attraction, and a lower activation energy is acquired to intercalate into the cathode interlayer. The Zn^2+^ diffusion coefficient determined from GITT also correlates with this reasonable illustration, for which a greater diffusion coefficient accompanied by the composite binder accelerates the hydrated Zn^2+^ desolvation and intercalation kinetics. Compositing PTFE with SA, an ≈40% lower activation energy eventually enables a high‐rate performance and higher specific capacities. It could be concluded that the hybrid binder facilitates the desolvation process for both Zn^2+^/H^+^ at the interface.

**Figure 4:**
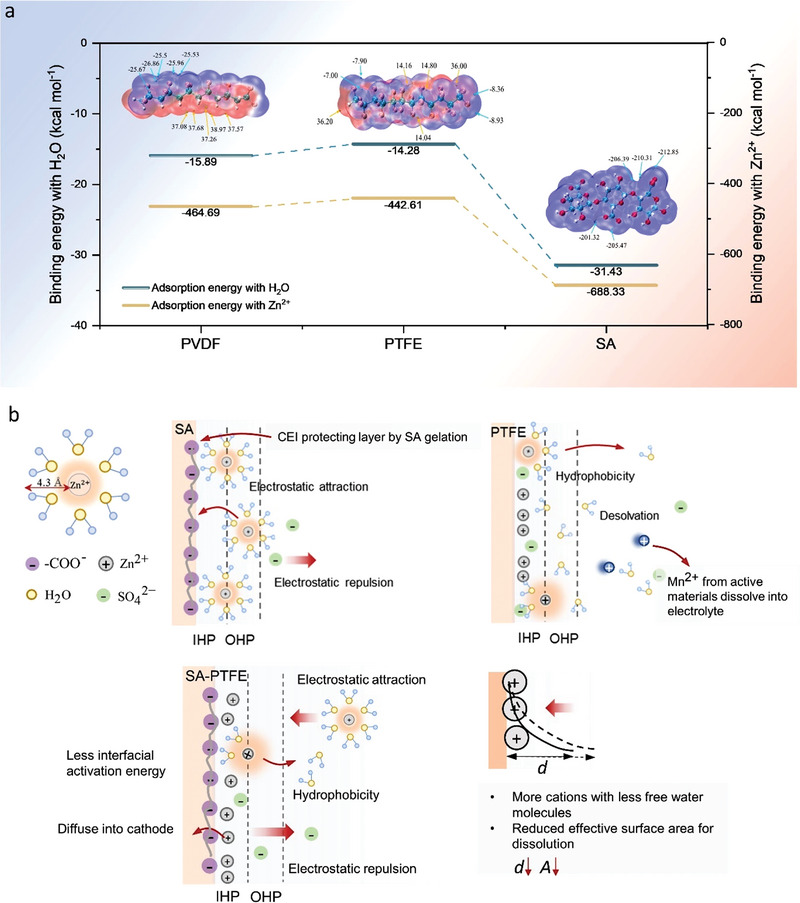
a) Binding energy for PVDF, PTFE, and SA with Zn^2+^ and H_2_O at reacting position P2 from the calculations; b) schematic diagram of the kinetics at the CEI for different binders. For pristine binder SA, hydrated Zn^2+^ ions are attracted to the interface by the affinity of SA, forming a CEI protecting layer. For PTFE, desolvation is facilitated by hydrophobicity, while there is no protecting layer to prevent the Mn^2+^ dissolution. The hybrid binder SA‐PTFE exhibits a CEI layer with more Zn^2+^ but less free water molecules. The less interfacial activation energy facilitates the Zn^2+^ diffusion.

DFT results have confirmed the binding energy between different binders and Zn^2+^ sheaths. Due to the repeated unit present in all binder components, five different reacting positions are selected on the backbone for PVDF, PTFE, and SA (Figure S13, Supporting Information). Typically, Zn^2+^ ions are coordinated by six water molecules in the first outer coordination shell, forming an octahedral shape of [Zn(H_2_O)_6_]^2+^. The desolvation mechanism affects the zinc diffusion ability, especially under a high current density. The balance of hydrophobic and hydrophilic moieties could accelerate the solvation effect and decrease the number of water molecules surrounding Zn^2+^ cations when Zn‐ions insert into the host materials, promoting Zn^2+^ transport and charge transfer.^[^ ^51^, ^52^ ^]^ **Figure** **4**a demonstrates the binding energies for these binders with H_2_O and Zn^2+^ at reacting position P2, and other calculated results are listed in Table S3, Supporting Information. Deduced from DFT calculation, the binding energies for one water molecule can be ranked as PTFE < PVDF < SA. With the application of pristine SA as the binder, the strong adsorption energy with water of −31.43 kcal mol^−1^ allows water molecules pass through the cathode‐electrolyte interface prohibiting the desolvation of [Zn(H_2_O)_6_]^2+^ to Zn^2+^,^[^ ^52^, ^53^ ^]^ thus resulting in a severe sluggish energy storage mechanism. Referring to electrochemical results, SA exhibits the lowest specific capacity in all current densities and a fast capacity decay compared to PVDF and PTFE. The adsorption energy of PTFE (−14.28 kcal mol^−1^) and a water molecule is similar to that of PVDF (−15.89 kcal mol^−1^), endowing the interface with hydrophobicity. Hydrophobic interactions at the interface interrupt hydrogen bonding between water and Zn^2+^ ions, facilitating the desolvation at the interface. As for hybrid binders with SA, the presence of hydrophobic PTFE in the hybrid binder accelerates the desolvation of hydrate Zn^2+^ ions. Consistent with the results of surface wettability (Figure S10a, Supporting Information), the contact angles increase with increasing amounts of PTFE in the composite, indicating increased surface energy at the CEI. Combined with electrochemical performances, it is surprising that specific capacity increase with the increase of cathode hydrophilicity in the hybrid binder, reaching an optimum composition in the volcano‐type profile as shown in Figure S10a, Supporting Information, where the contact angle is 104.91°. ZIBs with the P_1_S_4_ binder exhibit a superior specific capacity and capacity retention. As for ZIBs with the pristine SA exhibit the lowest specific capacity and capacity retention despite the best hydrophilic behavior. This further confirms that PTFE and SA composites could manipulate the desolvation energy, where a moderate hydrophobic ability could be achieved with adding PTFE in SA.

As for the interaction among binders and Zn^2+^ ions, binding energies are −684.98, −464.56, and −442.16 kcal mol^−1^ for SA, PVDF, and PTFE, respectively. Owing to the affinity of SA with Zn^2+^, the binding energy of SA and Zn^2+^ is expected to be the strongest compared to others. Subsequently, SA can be regarded as an anionic polyelectrolyte attracting Zn^2+^ ions forming a highly concentrated inner layer at the cathode–electrolyte interface, as shown in Figure 4b. Attribute to SA, the anionic layer formed at the CEI will attract Zn^2+^ by the strong electrostatic force, resulting in a higher rate of capacitive behavior. However, pristine SA also exhibits a strong combining capacity for water molecules, absorbing the Zn^2+^ sheath with more water content. Pristine PTFE exhibits superior desolvation capability, while there is no CEI layer to avoid the dissolution of Mn^2+^ from the cathode. Introducing PTFE to SA accelerates the desolvation process, for which partially hydrated Zn^2+^ ions are attracted at the interface, increasing the areal density of Zn^2+^ ions. Meanwhile, the hydroxyl‐terminated group from the SA backbone at the surface also facilitates the H^+^ intercalation through hydrogen bonding.^[^ ^8^ ^]^ The strong surface adsorption bonding of the hybrid binder with Zn^2+^ and H^+^ results in the intercalation mechanism from an aqueous medium in a partially hydrated regime.^[^ ^31^ ^]^ The high areal density of charge carriers leads to a significant concentration variation ($\frac{\partial C}{\partial x}$, *c* is the concentration, *x* is the distance) at the interface, thus contributing to a higher diffusion flux according to Fick's law ($J_{0}=-D_{0}\frac{\partial C}{\partial x}$). Considering the capacity from the EDLC, high Zn^2+^ areal density also increases the active surface area between the electrode and the electrolyte, hence improving surface chemical reactions, especially at a high current density. In the meantime, the concentrated region of Zn^2+^ formed at the CEI further restricts the dissolution. Referring to the diffusion‐dissolution model,^[^ ^13^ ^]^ the kinetics of the Noyes–Whitney equation ($\frac{dm}{dt}=A\frac{D}{d}\left(C_{s}-C_{d}\right)$, $\frac{dm}{dt}$ is the dissolution rate, *A* is the active surface area, *D* is the diffusion coefficient of pre‐intercalated Na^+^, *d* is the diffusion layer, *C*_s_ and *C*_d_ are concentrations at interface and diffusion layers, respectively), a lower active surface area between active materials and water molecules also reduce the dissolution rate. Tuning the electrode surface morphology from the binder, it is a universal approach to maintain a balanced competition between Zn^2+^ and H^+^ intercalation at the interface. With a stronger affinity to Zn^2+^, pristine SA is capable to attract Zn^2+^ at the CEI interface, however, the accompanied water molecules are not beneficial for desolvation at the interface. PTFE and PVDF exhibit strong hydrophobicity for the desolvation process, while the lack of an anionic layer at the CEI interface eventually results in a less capacity at the high current density. Introducing PTFE into SA, both merits of desolvation and anionic attracting layer at CEI furthermore screen the Coulombic repulsion of the Zn^2+^ ions during intercalation and result in a high Zn^2+^ diffusion coefficient and low activation energy, hence all hybrid binders exhibit superior GCD performance, especially at high current densities.

**Figure 5:**
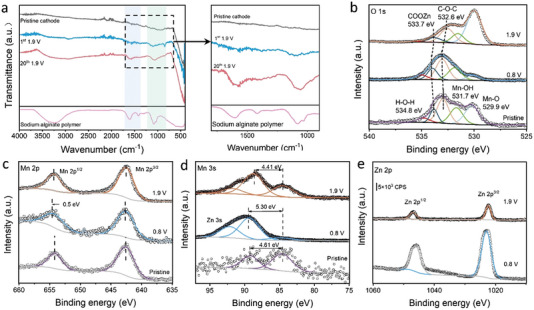
a) Ex situ FTIR for the cathode with the hybrid binder P_1_S_4_ at charged states; b–e) Ex situ XPS for the cathode with the hybrid binder P_1_S_4_ of O 1s, Mn 2p, Mn 3s, and Zn 2p respectively.

Ex situ XPS, FTIR and XRD characterizations were performed to investigate the phase variation of the cathode with the P_1_S_4_ hybrid binder. Ex situ FTIR was used to further verify the in situ activation process of CEI. As shown in **Figure** **5**a, at the charged state, the appearance of Zn_4_SO_4_(OH)_6_∙5H_2_O overlaps the characteristic bands of the alginate, while it disappears at the discharged state. Increasing intensity at the bands of 1585 and 1070 cm^−1^ further confirms the in situ gelation of CEI during cycling. As shown in Figure S11, Supporting Information, ex situ XRD spectra were measured at the potentials where the plateaus occur from 1st to 30th cycles under 0.1 A g^−1^, and there is a change in the characteristic phases. During discharging, Zn^2+^ ions inserted into the cathode, Zn_4_SO_4_(OH)_6_∙5H_2_O was observed at both discharged states of 0.8 V for 1st, 7^th^, and 30th cycles, while it disappeared during charging from 0.8 to 1.9 V. The reversible formation of Zn_4_SO_4_(OH)_6_∙5H_2_O not only revealed reversible Zn^2+^ insertion/extraction but also proved the formation of MnOOH, whereby Mn^4+^ was reduced to Mn^3+^. Ex situ XPS (Figure 5b–e) showed the variation of the average valence states (AOS) during cycling. O 1s spectra can be identified as Mn—O (529.9 eV), Mn—OH (531.7 eV), and H—O—H (534.8 eV).^[^ ^32^, ^46^ ^]^ Carboxylic groups that used for CEI protective layer formation is located at 533.7 and 532.6 eV.^[^ ^16^, ^54^ ^]^ Peak shifts in COOZn indicate the coordination of —COO^−^ with Zn^2+^ during discharging. The Mn 3s peak difference is 5.30 eV at the discharged state and 4.1 eV at the charged state, corresponding to AOS = 2.99 and AOS = 3.97, respectively, in accordance with AOS = 8.95 − 1.13∆*E*,^[^ ^50^, ^55^, ^56^ ^]^ where ∆*E* stands for the energy difference between the main Mn 3s peak and its satellite peak. Considering the disproportionation of Mn^3+^, the formed CEI inhibits the change from Mn^3+^ to Mn^2+^, hence enhancing the stability of the cathode with higher capacity retention. In terms of the Mn 2p spectra, doublet peaks located at the binding energy of 654 and 642 eV are assigned to Mn 2p^1/2^ and 2p^3/2^, respectively. Owing to the reversible insertion/extraction of H^+^/Zn^2+^, the binding energy at the discharged state shifts to 654.68 eV from the original state of 654.18 eV, while it recovers to the pristine state at the charged state. Compared to the ex situ XPS results for the cathode using conventional binder PVDF (Figure S14, Supporting Information), average oxidation states attained at discharged and charged states are AOS = 2.67 and AOS = 3.51 respectively, which are less than the one for the hybrid binder. Hence, without the presence of protecting CEI layer, less stable Mn^3+^ ions will be further disproportionated to Mn^2+^ resulting in a severe disproportion reaction for cathodes with the PVDF binder. To further verify the feasibility of the hybrid binder with other cathode materials, the cathode composed of commercial MnO_2_ and the hybrid binder was also investigated. As shown in Figure S12, Supporting Information, consistent with previous results, the ZIB with the hybrid binder exhibits a higher surface‐controlled contribution and better rate performances than the one with the PVDF binder. A higher surface‐controlled contribution leads to superior rate performance. The ZIB with the hybrid binder even possesses a specific capacity of 144% greater than the PVDF one at a current density of 5 A g^−1^.

## 3 Conclusion

In summary, a universal strategy was established in this work to stabilize cathode materials by using well‐designed binder components. A water‐soluble binder was developed by forming a composite of polysaccharide alginate and hydrophobic PTFE. With increasing sodium alginate content in the composite, there is an increased contribution from surface‐controlled charge storage processes, resulting in an increased rate performance, especially at high current densities. A balance of PTFE and SA subsequently results in an optimal ratio as the hybrid binder, P_1_S_4_ (PTFE: SA), exhibits an average ZIB‐specific capacity of 149.8 mAh g^−1^ at 2 A g^−1^, 45.6% greater than the one with commercial PVDF. An intensive investigation was performed to understand both kinetic and thermodynamic behaviors. This showed that lower activation energy (16.57 kJ mol^−1^) and a higher diffusion coefficient benefit from attaining a high ZIB capacity. DFT calculations further unveil the mechanism of the hybrid binder, whereby the sodium alginate is acting as an anionic polyelectrolyte, accelerating the Zn^2+^ diffusion from the electrolyte to the cathode. Moreover, with the advantage of hydrophobic PTFE and the fast desolvation process, a high areal density of Zn^2+^ at the interface can be obtained. Apart from the surface morphology manipulation, the hybrid binder also helps to form and protect the CEI layer. The in situ formed CEI inhibits the disproportion of Mn^3+^, hence contributing to improved stability. This universal strategy provides a promising approach to modifying the CEI interface for aqueous batteries from a cost‐effective binder recipe.

## 4 Experimental Section

Data for all the electrochemical tests were examined for at least three samples for each binder composition. Data for Arrhenius test was processed by logarithm. All the data for material characterizations such as XRD, and FTIR were proceeded by OriginLab from the text file exported from the testing devices. XPS data was analyzed by CasaXPS. The adhesion test was conducted by calculating the number of pixels from the images by a MATLAB code with the function of image processing.

## Conflict of Interest

The authors declare no conflict of interest.

## Supporting information

Supporting Information

## Data Availability

The data that support the findings of this study are available from the corresponding author upon reasonable request.
