# Supplementary material for: Cathode–Electrolyte Interface Modification by Binder Engineering for High‐Performance Aqueous Zinc‐Ion Batteries
Source: Adv Sci (Weinh). 2022 Dec 16;10(5):2205084. doi: 10.1002/advs.202205084 (PMC9929112; doi:10.1002/advs.202205084)
Supplement: Supplementary file 1 — Supporting Information [file ADVS-10-2205084-s001.pdf]

ADVANCED SCIENCE
Open Access

Supporting Information

for *Adv. Sci.*, DOI 10.1002/advs.202205084

Cathode–Electrolyte Interface Modification by Binder Engineering for High-Performance Aqueous Zinc-Ion Batteries

Haobo Dong, Ruirui Liu, Xueying Hu, Fangjia Zhao, Liqun Kang, Longxiang Liu, Jianwei Li, Yeshu Tan, Yongquan Zhou, Dan J.L. Brett, Guanjie He and Ivan P. Parkin**

Supporting information

Chemicals

All chemicals were used as purchased without further purification. Polytetrafluoroethylene (PTFE) preparation (60 *wt.*% dispersion in H_2O) (Sigma-Aldrich); poly(vinylidene fluoride) (PVDF), average Mw ~534,000 by powder (Sigma-Aldrich); 1-Methyl-2-pyrrolidinone, 99% (Sigma-Aldrich); manganese (II) nitrate tetrahydrate, 99% (Sigma-Aldrich); Sodium hydroxide, reagent grade, 97%, powder (Sigma-Aldrich); hydrogen peroxide solution 30 % (w/w) in H_2O (Sigma-Aldrich); alginic acid sodium salt from brown algae (SA), medium viscosity (Sigma-Aldrich); manganese(II) sulfate monohydrate (≥99%) (Sigma-Aldrich); zinc sulfate heptahydrate (≥99%) (Sigma-Aldrich).

Synthesis of polymer binders

The hybrid binder was synthesized by mixing SA and PTFE in the solvent of deionized water (D.I. water). SA solution was prepared initially by dissolving 4 g SA into 20 mL D.I. water (2 mg in 10 μL) followed by stirring for 24 hours. A light brown solution was obtained after SA was homogeneously dispersed in the solvent. As for PTFE, 60 *wt.*% dispersed PTFE in H_2O was utilised. Different compositions of PTFE and SA were prepared for hybrid binders with short names as P_4S_1, P_1S_1, and P_1S_4 refer to the PTFE:SA weight ratios of 4:1, 1:1 and 1:4, respectively. The extra D.I. water was added into the solution in the concentration of 0.5 g per 10 mL. An additional 3-hour stirring was applied to the hybrid complex allowing a uniform mixture of both solutions. As for the comparison, conventional binder PVDF was also prepared by dissolving the PVDF powder into a NMP solution with concentration of 5 wt.% (5 mg in 100μL).

Synthesis of cathode materials

Na-Birnessite MnO_2 was synthesised by a facile co-precipitation method. The solution A and B were prepared by dissolving 15 mmol of manganese (II) nitrate tetrahydrate $Mn(NO_3)_2 \cdot 4H_2O$ and 55 mmol of NaOH into 50 mL of D.I. water and a mixed solution of 12 mL of H_2O_2 (30 *wt*%) and 90 mL of D.I. water, respectively. Both the solutions were stirred under ambient environment for 10 minutes, and then, the solution B was quickly poured into solution A with vigorous magnetic stirring for another 10 minutes. It is seen that the black precipitate in solution was formed immediately, and the solution was preserved in an ice bath for 24 hours afterwards. The final product was collected and washed by distilled water for 5 times and dried under freeze-drying process.

Electrode preparation

The electrode was prepared with the conductive agent Super P and the binder with the mass ratio of 7:2:1, followed by casting the cathode slurry onto a carbon paper from Toray UK (Toray TGP-H-60). For coherence and consistency, the same batch of current collectors and active materials were used for cathode fabrication. It is noted that the binder is thoroughly agitated prior to cathode casting procedure.

Material characterisations

X-ray diffraction (XRD) patterns were examined by a STOE SEIFERT diffractometer with detected angular range of $2° < 2\theta < 45°$ under the radiation source of metal Mo. X-ray photoelectron spectroscopy (XPS) were obtained from Thermo scientific K-alpha photoelectron spectrometer and analysed by CasaXPS. Scanning electron microscope (SEM) and transmission electron microscopy (TEM) were conducted by Carl Zeiss EVO MA10 and JEOL-JEM-2100, respectively. Scanning transmission electron microscope (STEM) (JEOL ARM300CF, beamline E02 of Diamond Light Source (UK). Fourier-transform infrared spectroscopy was measured by Shimadzu IRTracer-100 from the wavenumber from 400 to 4000 cm^{-1}. The mass of the active materials was weighed accurately by an analytical balance (Ohaus; δ= 0.01 mg). Raman Spectroscopy was examined by Renishaw Raman microscope spectrometer in a laser wavelength of 514.5 nm. Surface wettability was examined by an

optical contact angle meter via the sessile-drop approach at ambient environment (FTA 1000). The electrolyte droplets were kept in a constant volume of 5 μL and dwelling time of droplets stayed on each sample were set up to 60 s. A program-controlled furnace (NEWARE: MGDW-150-20H) with temperature range -40-150℃ was utilised to investigate the EIS spectra under different temperatures.

Electrochemical characterisations

Battery galvanostatic charge-discharge tests were measured by NEWARE battery testing systems. Cyclic Voltammetry (CV) and Electrochemical Impedance Spectroscopy (EIS) were investigated by the VMP3 Biologic electrochemical workstation and Gamry 1010E, respectively. CV was measured in sweep rates from 0.1 to 5 mV s^{-1}. EIS was measured from 10^5 to 0.1 Hz. Galvanostatic Intermittent Titration Technique (GITT) was applied to determine the Zn^{2+} ion diffusion coefficients by NEWARE battery testing systems.

GITT analysis

GITT was utilised to investigate the Zn^{2+} diffusion coefficient for ZIBs with both P_1S_4 and commercial PVDF binders. The test was performed at a galvanostatic pulse of 50 mA g^{-1} for 20 min followed by a 120 min relaxation process to reach equilibrium. This pulse-relaxation procedure was repeated during charging/discharging process to 1.8/0.9 V. Subsequently, the Zn^{2+} diffusion coefficient can be calculated by the following equation:

$$D_{Zn} = \frac{A}{\pi}(\frac{m_b V_M}{M_b A})^2(\frac{\Delta E_s}{\tau(dE_\tau/d\sqrt{\tau})})^2 , (\tau \ll L^2/D_{Zn})$$

Where D_{zn} represents the diffusion coefficient, τ is the duration time of the current pulse, m_b is the mass of the active material, M_b and V_m are the molecular weight (g mol^{-1}) and molar volume (cm^3 mol^{-1}) respectively, A is the total contacting area of the electrode with the electrolyte, $dE_\tau/d\sqrt{\tau}$ is the slope of the linear region of the potential E_τ during a current pulse in the duration time τ, ΔE_s is the difference in the open circuit voltage measured at the end of the relaxation period for two successive steps, and L is the thickness of the electrode. If the variation of cell voltage ΔE_τ during titration was in a linear relationship against $\tau^{1/2}$, the above equation is derived as:

$$D_{Zn} = \frac{A}{\pi\tau}(\frac{m_b V_M}{M_b A})^2(\frac{\Delta E_s}{\Delta E_\tau})^2$$

Arrhenius theory

Besides kinetic analysis, thermodynamic analysis was evaluated according to Arrhenius theory where the interface activation energies were calculated between commercial PVDF and the hybrid binder P_1S_4. As stated in the argument that a minimum energy acquired for reactants to transform into products is temperature dependence following the Arrhenius equation as expressed below:

$$k = Ae^{\frac{-E_a}{RT}}$$

where k refers to the reaction rate constant, A is a constant parameter of pre-exponential factor, E_a is the activation energy, and R and T are universal gas constant and absolute temperature respectively. After the logarithm calculation, the formular can be derived as below. To exploit further insight into the interfacial charge transfer process, EIS spectra were recorded in the temperatures range of 30–70℃ for AZIBs with 3M $ZnSO_4$ and 0.2 M $MnSO_4$ electrolyte. In this case, rate constant k is the reciprocal of R_{ct} extrapolating from the impedance data at different temperatures, hence the activation temperature can be derived from the gradient of the linear slope as expressed in the following equation. Referring to aforementioned variations in charge transfer impedances before and after GCD, R_{ct} was selected to evaluate the interfacial kinetics.[1,2]

$$lnk = lnA - \frac{E_a}{RT}$$

$$\text{hence, } \ln\left(\frac{1}{R_{ct}}\right) = lnA - \frac{E_a}{RT}$$

DFT simulation

DFT calculations were conducted to corroborate chemical interactions between different binders and Zn^{2+} sheaths with the aim to unveil the mechanism at the cathode-eletrolyte interface with different binders. To explain the different performances of the hybrid binder SA-PTFE to PVDF and PTFE, the DFT calculation was performed to investigate the binding energy of water molecule and zinc ion in different binding sites of binders SA, PVDF and PTFE. The initial structure of SA, PVDF and PTFE was prepared using GaussView 6.0, and all calculations were done with Gaussian09[3]. The aug-cc-pvdz basis set was used for all atoms. To reduce the cost of computation, three units of SA and six units of PVDF and PTFE are used in computational part. Then, all wavefunction analyses were conducted by Multiwfn 3.5[4]. The binding energy can be defined as:

$$E_{binding} = E_{total} - E_{base} - E_{tar}$$

where $E_{binding}$, E_{total}, E_{base}, and E_{tar} correspondingly devote to the binding energy, the total energy of the adsorption model, the energy of the base such as PTFE, PVDF and SA, and the energy of the target such as water molecules and Zn^{2+} ions.

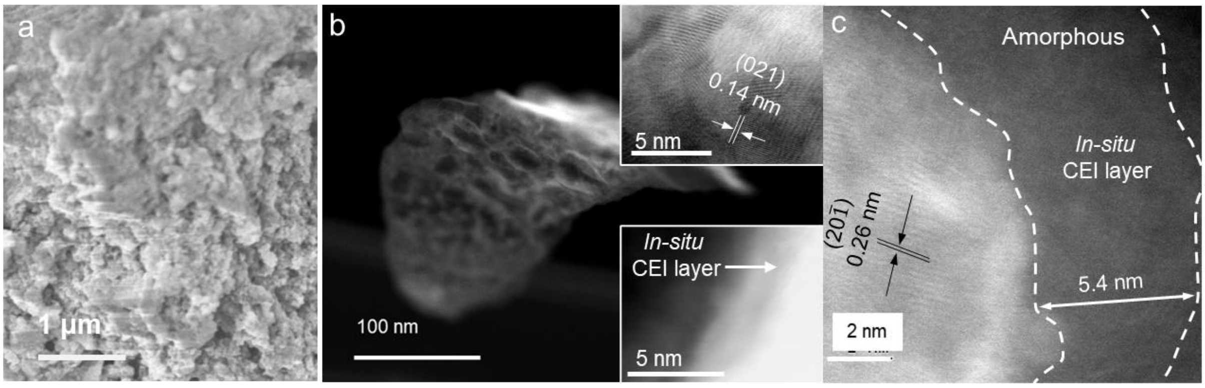

Figure S1 (a) SEM image for cathode with the PTFE-SA binder before cycling; (b)STEM images for the cathode with the PTFE-SA binder after 30 cycles at 0.1A g^{-1}. The porous structure indicates the formation of the sodium alginate polymer structure. The inner pictures are zoomed regions at the inside and the edge, respectively; (c) STEM images for the cathode with the PTFE-SA binder after cycling at the edge where the amorphous region, *in-situ* CEI layer, surrounds the MnO_2.

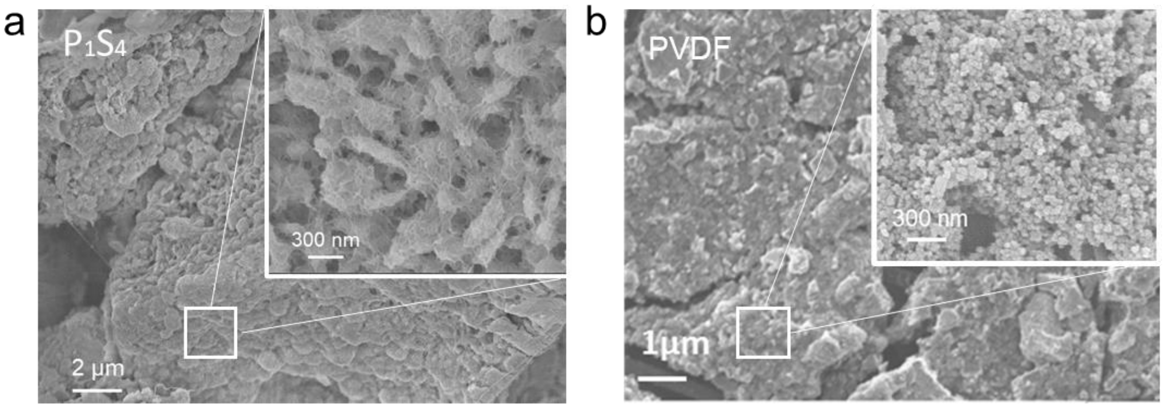

Figure S2 SEM images of surface morphology comparison for (a) hybrid binder and (b) PVDF.

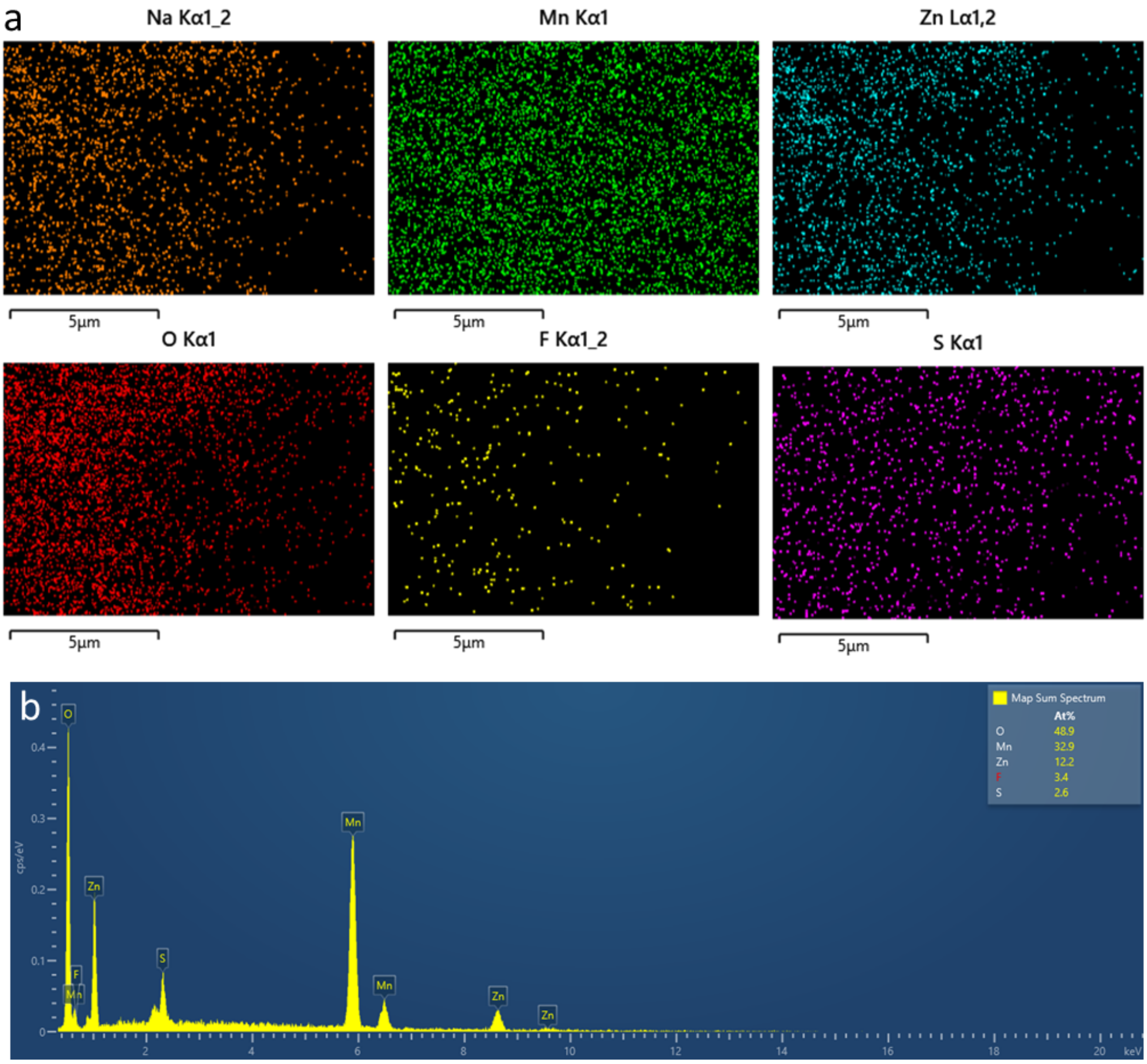

Figure S3 (a) EDS mapping of the sample after cycling (corresponding to Figure 2c); (b) elemental ratios.

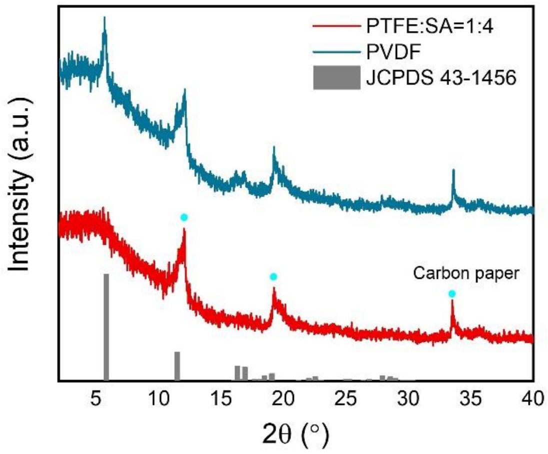

Figure S4. XRD spectra cathodes with different binders

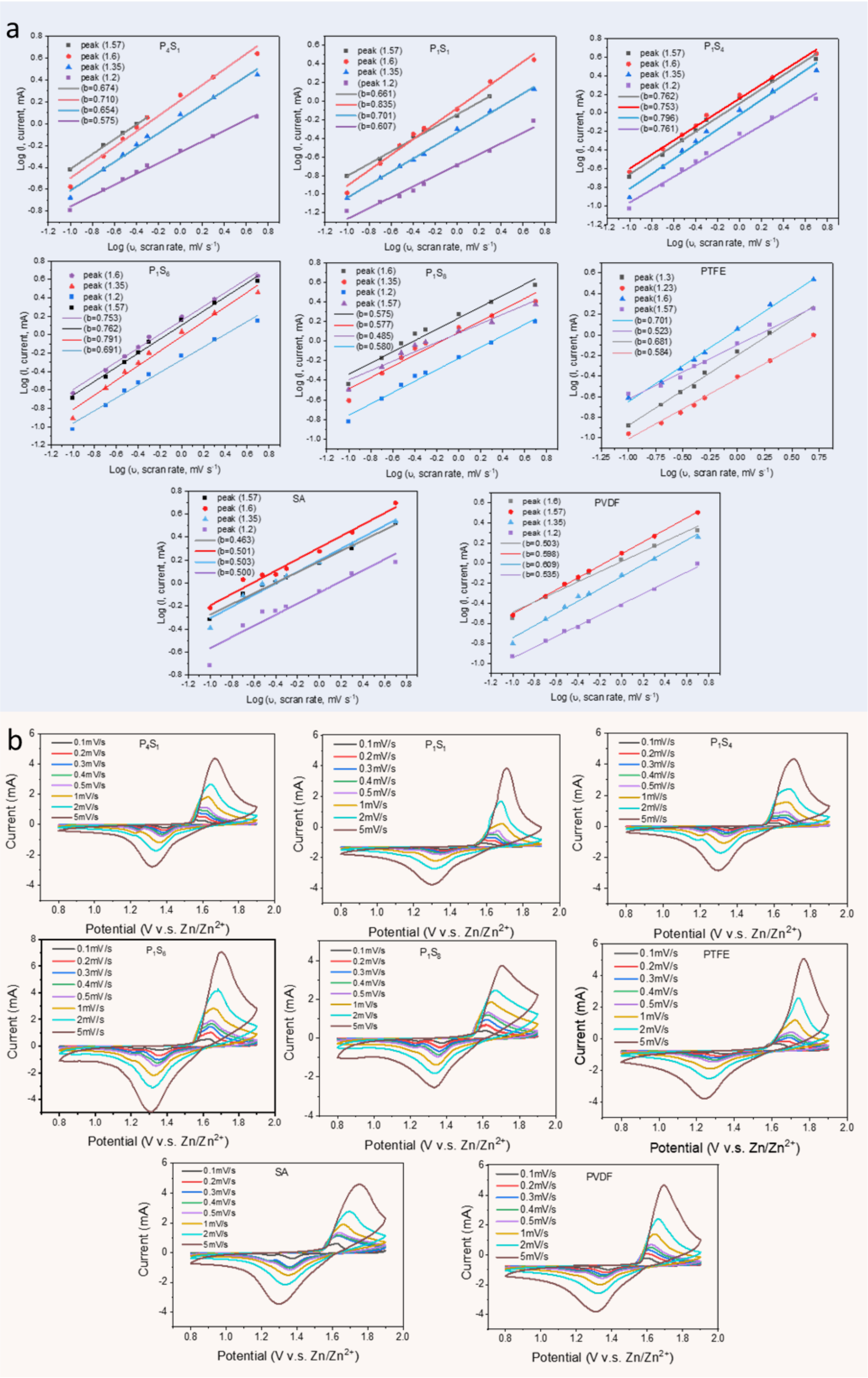

Figure S5 (a) CV profiles for ZIBs with different binders; (b) Calculated *b* values for ZIBs with different binders.

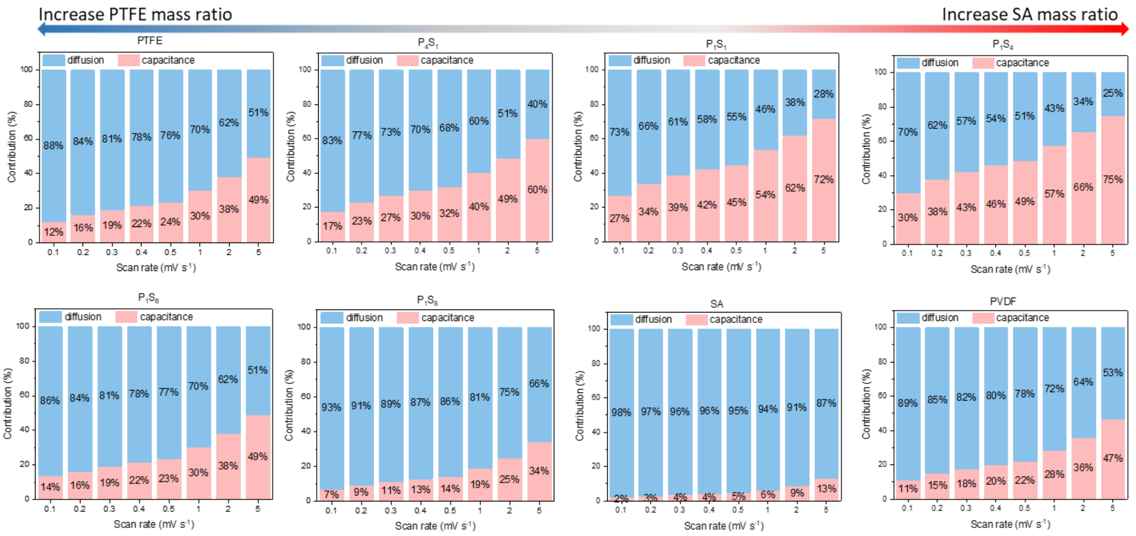

Figure S6 Contribution of diffusion and capacitive control of different binders with the increasing mass ratio of SA in the composites.

Table S1 Resistance calculated from corresponding Nyquist plots for different binders

	Before			After		
	R_b (Ω)	R_f (Ω)	R_{ct} (Ω)	R_b (Ω)	R_f (Ω)	R_{ct} (Ω)
PVDF	1.16		55.19	5.57		53.92
PTFE	2.60	68.09	91.95	3.56	9.87	29.75
P_4S_1 (4:1)	3.64	96.51	102.31	5.19	78.05	30.03
P_1S_1 (1:1)	2.39	84.49	133.02	3.79	24.68	20.73
P_1S_4 (1:4)	2.61	57.11	258.09	4.23	5.88	21.31
P_1S_6 (1:6)	1.24	47.13	378.22	3.67	2.52	48.67
P_1S_8 (1:8)	1.93	53.02	395.13	1.36	4.52	68.51
SA	1.90	61.25	513.01	3.04	5.92	69.42

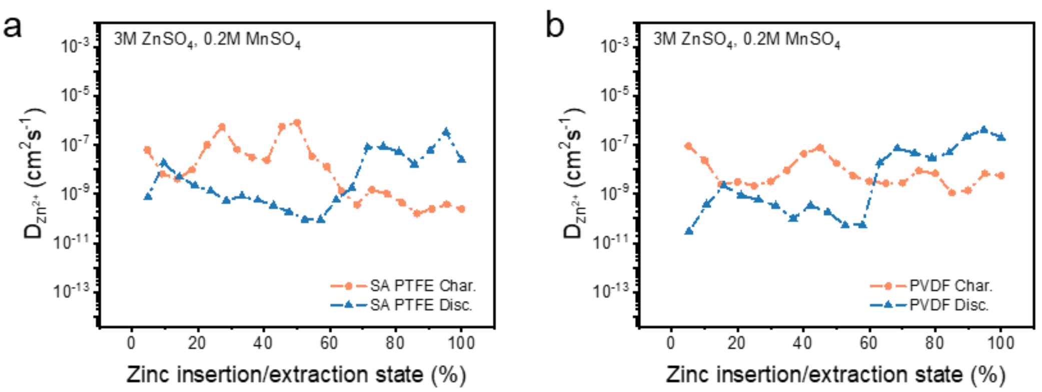

Figure S7 GITT for AZIBs with hybrid binder P_1S_4 and PVDF in the 3 M $ZnSO_4$ and 0.2 M $MnSO_4$ electrolytes. (a) P_1S_4 (b) PVDF.

Table S2 Average Zn^{2+} diffusion coefficient for hybrid and commercial binders.

Electrolytes	3M $ZnSO_4$ 0.2M $MnSO_4$	
	P_1S_4	PVDF
Average diffusion coefficient (cm^2 s^{-1})	6.79×10^{-8}	4.01×10^{-8}

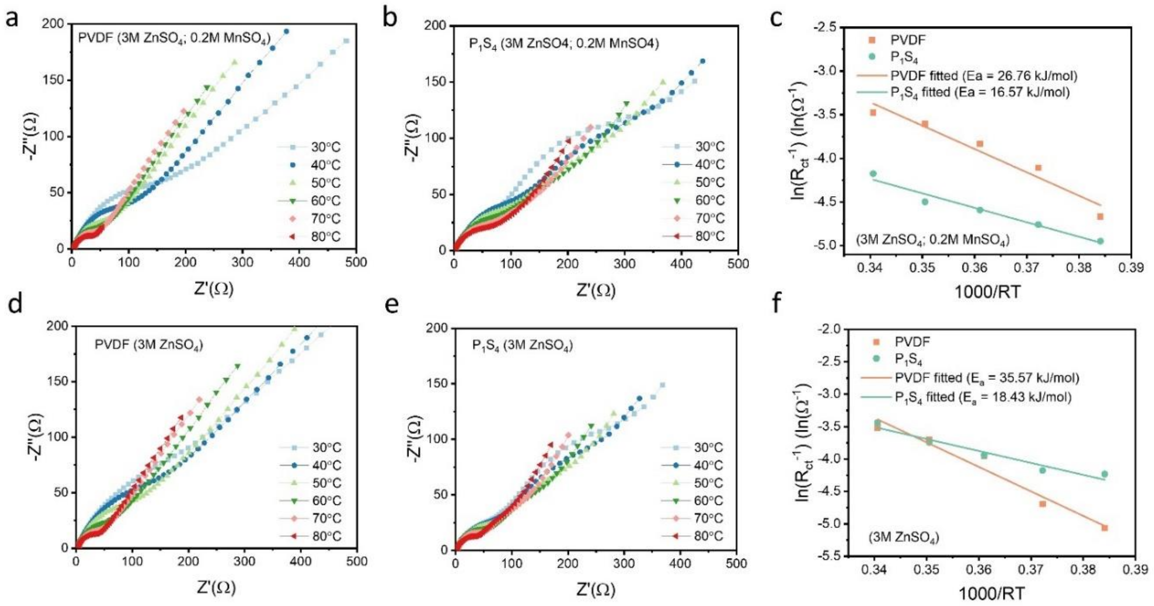

Figure S8 Activation energy check under electrolyte 3M $ZnSO_4$ and 0.2M $MnSO_4$. (a) Nyquist plots of PVDF; (b) Nyquist plots of P_1S_4; (c) activation energies for interfacial charge transfer. Activation energy check under electrolyte 3M $ZnSO_4$. (d) Nyquist plots for PVDF; (e) Nyquist plots of P_1S_4; (f) activation energies of interfacial charge transfer.

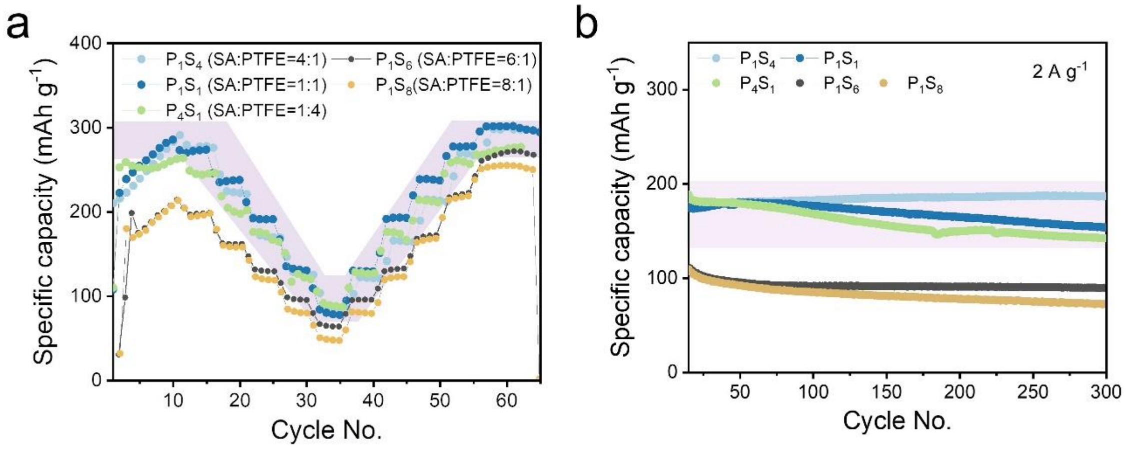

Figure S9 Galvanostatic charging/discharging performance. (a) Rate performance under current densities of 0.1, 0.2, 0.5, 1, 2, 5 A g^{-1} for ZIBs with the hybrid binders; (b) Long-term cycling performances for ZIBs with the hybrid binders at 2 A g^{-1}.

Surface wettability

The surface wettability is also a determinant to desolvation process. PTFE is a hydrophobic binder whereas SA is hydrophilic, hence for different compositions between hydrophilic and hydrophobic polymers, the surface wettability could be manipulated at the CEI interface. Contact angles were evaluated as indicated in the experimental section under a constant volume of 5 μL of aqueous electrolyte to the prepared cathode casted on the carbon paper. Figure S10 summarises the measured contact angles of cathode with hybrid binders and commercial binder PVDF respectively. It is noted that as for the pristine current collector, carbon paper, it exhibits a contact angle of 122.27°. While with an increased content of SA, the contact angle of the cathode fabricated decreases from 127.73° to 100.46°. PVDF also demonstrates a hydrophobic behaviour exhibiting a contact angle of 125.63°, which is consistent with previous reports[5,6].

Taking the electrochemical performance into consideration, it is surprising that specific capacity increase with the increase of cathode hydrophilicity in the hybrid binder reaching an optimum composition in the parabola profile as shown in Figure b, where the contact angle is 104.91°. Particularly, P_1S_4 binder exhibits a superior specific capacity and capacity retention. While as for pristine SA, it exhibits the lowest specific capacity and capacity retention despite the highest hydrophilic behaviour. To unveil the kinetic behaviour of the Zn^{2+} diffusion, a DFT calculation was carried to verify the desolvtion mechanism at the CEI.

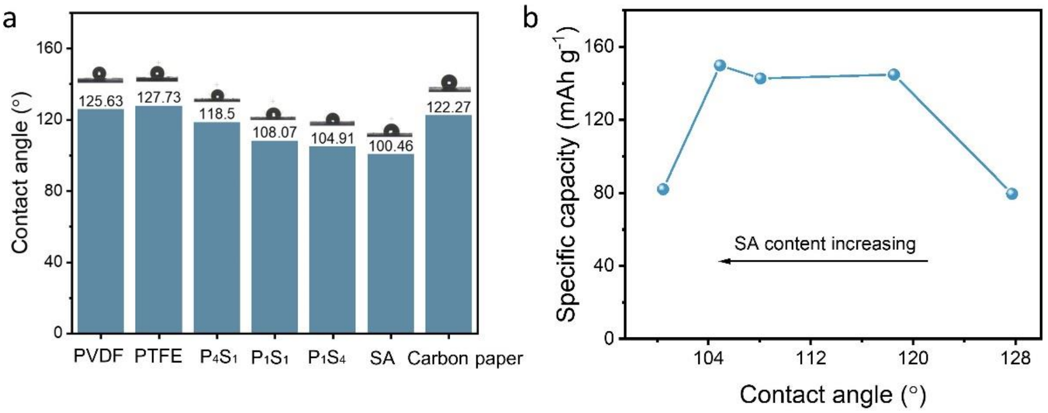

Figure S10 (a) Contact angle for different binders; (b) relation between specific capacity at 2 A g^{-1} with contact angles.

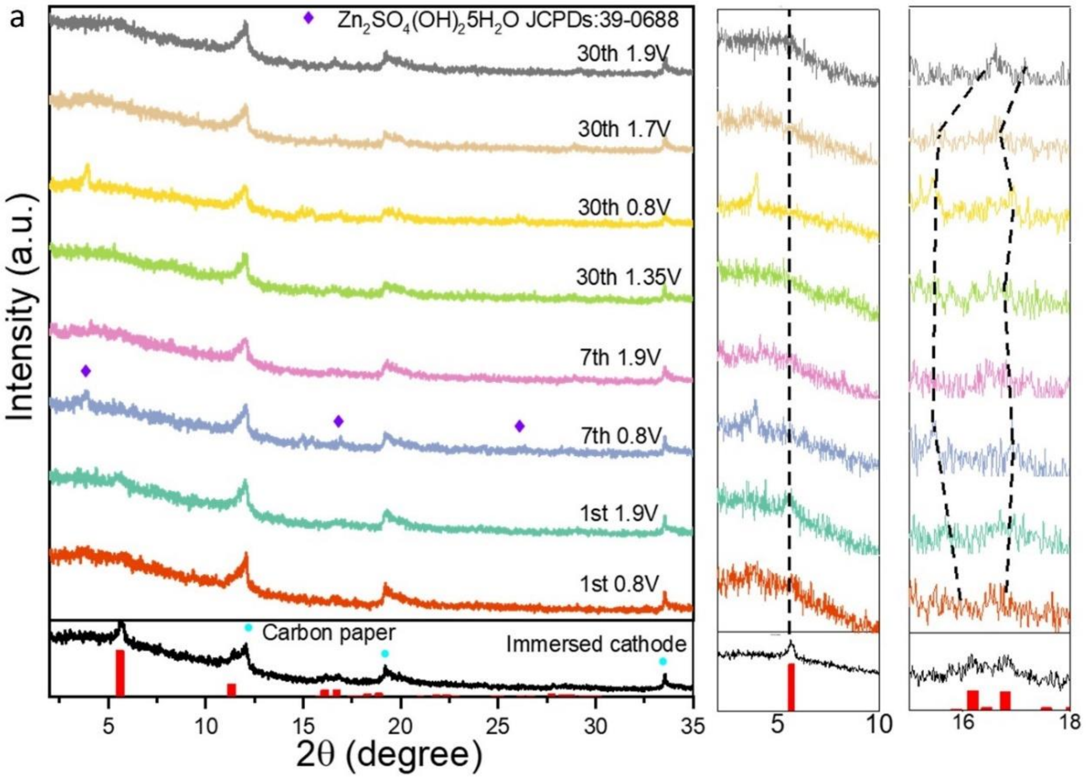

Figure S11 *Ex-situ* XRD for the cathode with binder P_1S_4 at different charged states.

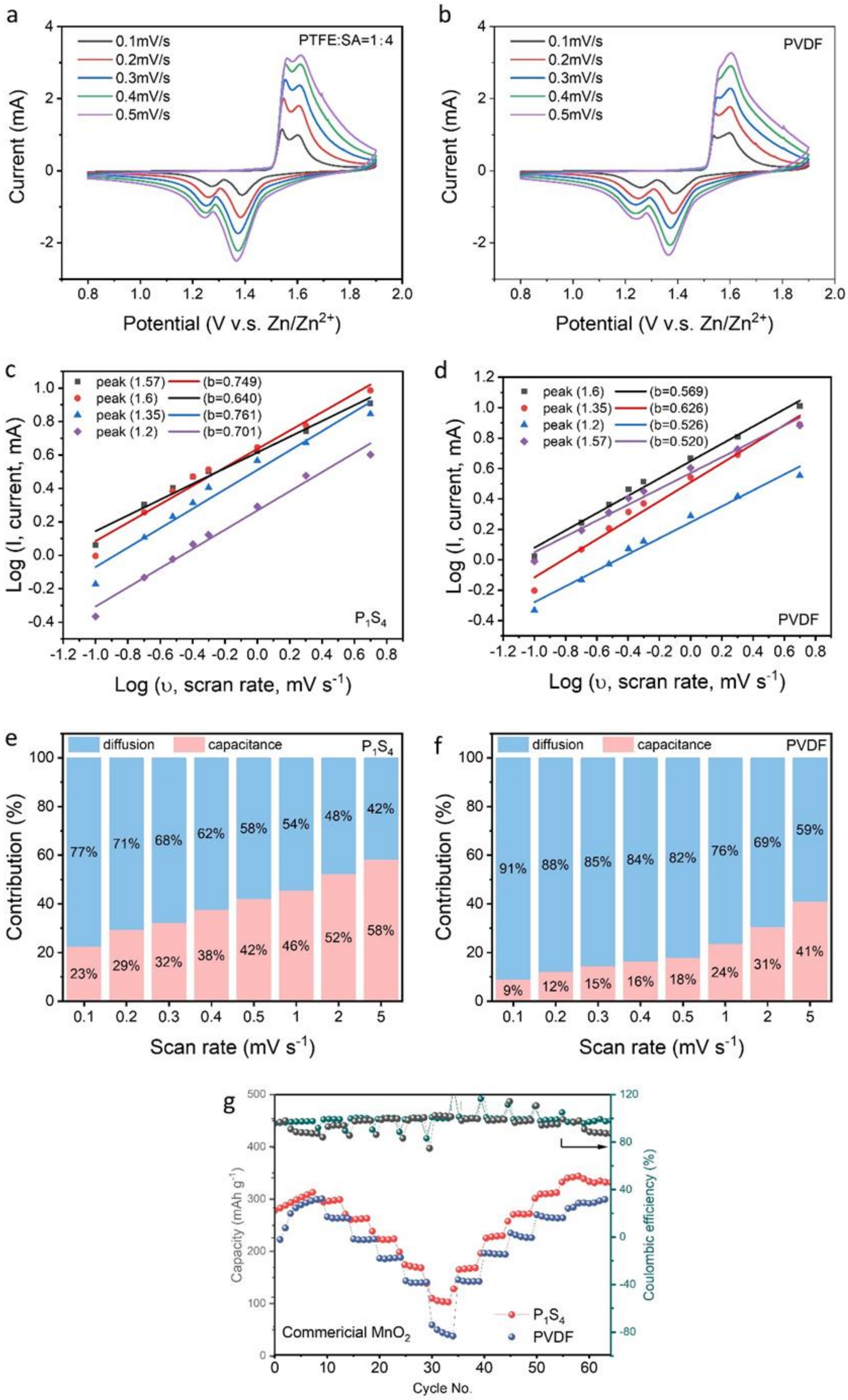

Figure S12 Diffusion controlled analysis for commercial MnO_2. (a),(c),(e) are CV of for hybrid P_1S_4 binder. (b),(d),(f) are CV test of PVDF binder. (g) GCD rate test.

Table S3 Binding energy for SA, PTFE and PVDF with H_2O and Zn^{2+}

	With single H_2O				
Binding energy	P1	P2	P3	P4	P5
PTFE (kcal/mol)	-16.98	-14.28	-14.28	-13.97	-16.94
SA (kcal/mol)	-	-31.43	-	-	-
PVDF (kcal/mol)	-18.78	-15.89	-18.96	-18.5	-15.77
	With Zn^{2+}				
PTFE (kcal/mol)	-442.16	-442.61	-443.59	-443.22	-
SA (kcal/mol)	-684.98	-688.33	-681.51	-677.32	-
PVDF (kcal/mol)	-464.56	-464.69	-464.69	-464.69	-464.38

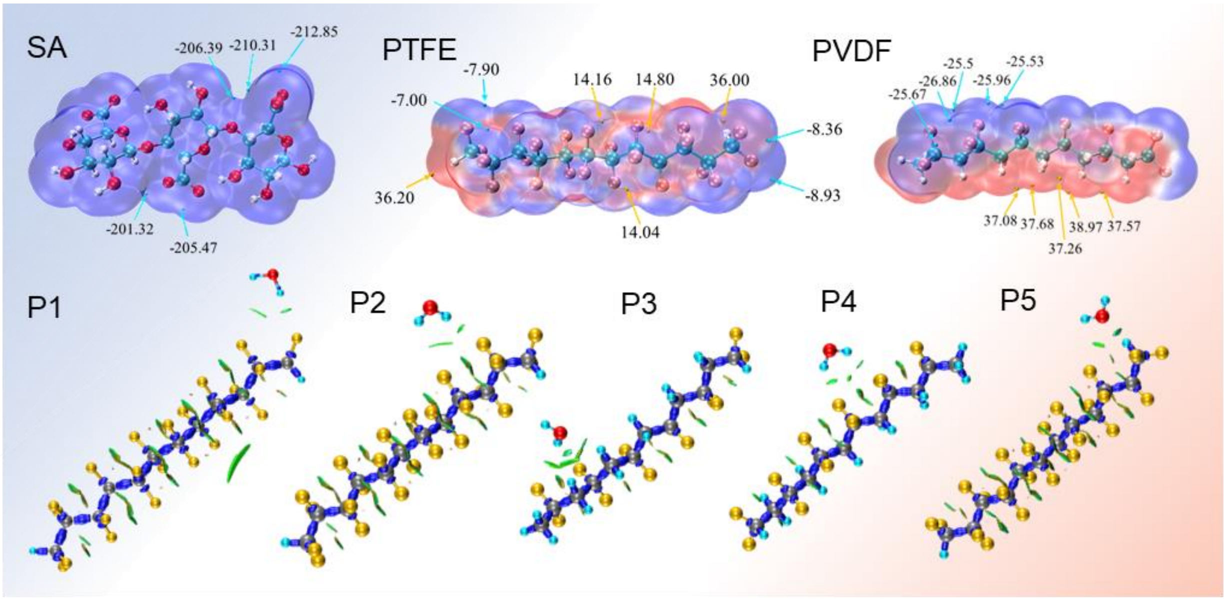

Figure S13 Electrostatic potential mapping and schematic diagram of reacting position from calculations. The example is for PTFE with one water molecule

Table S4 Cost comparison to commercial binder PVDF

	PVDF	NMP	PTFE	SA	D.I. Water
Cost $/Ton	74492.35	5959.39	11419.44	5000.00	1850.00
	PVDF/NMP (5wt%)		P4S1	P1S1	P1S4
Cost for binder $/Ton	9386.04		3507.11	3121.94	2736.78

Characterisations for cathode with binder PVDF

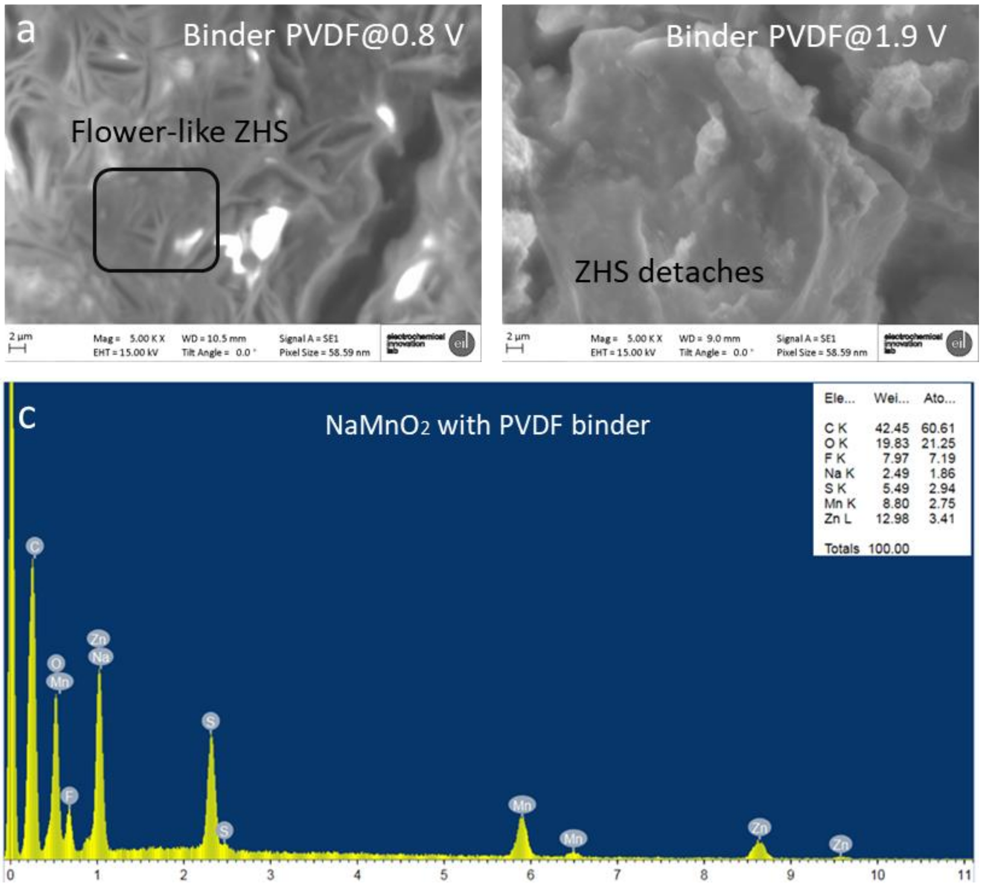

Figure S14 (a) SEM for $NaMnO_2$ with PVDF binder at 0.8 V; (b) SEM for $NaMnO_2$ with PVDF binder at 1.9 V (c) EDS results for $NaMnO_2$ with PVDF binder at 0.8 V.

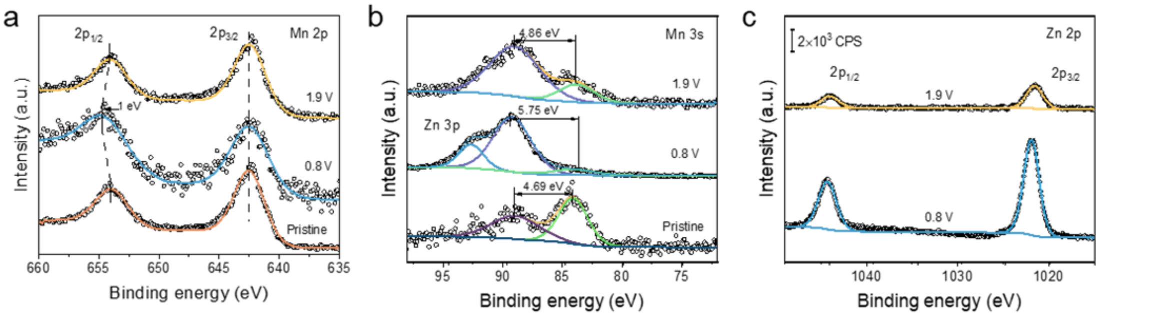

Figure S15 *Ex-situ* XPS spectra for $NaMnO_2$ with the PVDF binder. (a) Mn 2p; (b) Mn 3s; (c) Zn 2p at fully charged and discharged states.

Adhesion analysis

Digital image processing was conducted for the post analysis, where the active materials shown in the image was processed by the "blackwhite" functions in MATLAB. By calculating the pixels for the 200x200 image (see Figure S15), tape for P_1S_4 contains fewer active materials than the one for PVDF, which are 4840 and 8743 pixels respectively. Therefore, the hybrid binder exhibits a superior adhesion between the substrate and the active materials.

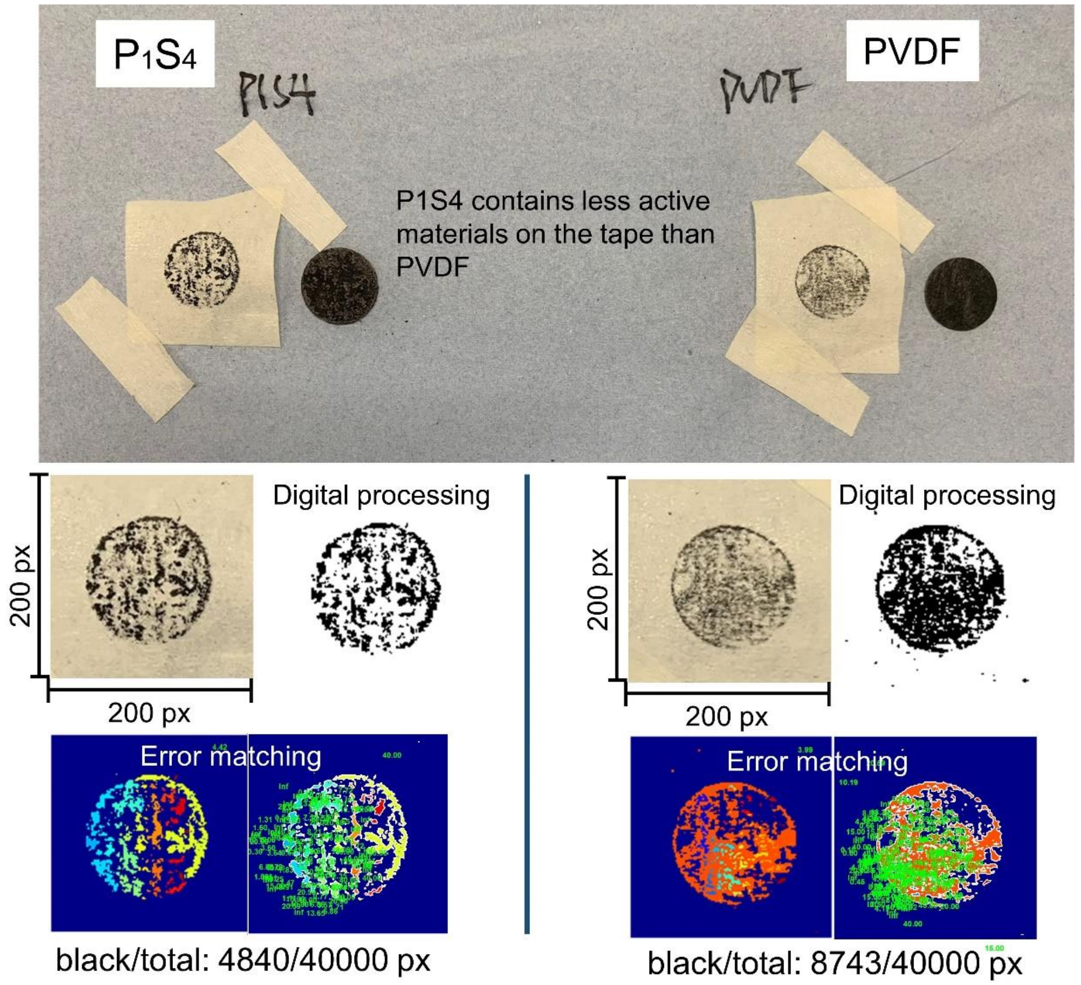

Figure S15 Binder adhesion comparison using an adhesive tape. The image is analysed by MATLAB image processing. Active materials on the tape for P_1S_4 is 4840/4000 pixels, while for PVDF active material is in the ratio 8743/40000 pixels.

Reference

[1] T. P. Heins, N. Harms, L. S. Schramm, U. Schröder, *Energy Technol.* **2016**, *4*, 1509.

[2] D. Kundu, S. Hosseini Vajargah, L. Wan, B. Adams, D. Prendergast, L. F. Nazar, *Energy Environ. Sci.* **2018**, *11*, 881.

[3] M. A. Frisch, M. J. E. A.; Trucks, G. W.; Schlegel, H. B.; Scuseria, G. E.; Robb, **2009**, 28.

[4] T. Lu, F. Chen, *J. Comput. Chem.* **2012**, *33*, 580.

[5] M. R. Mohd Ramli, A. L. Ahmad, C. P. Leo, *ACS Omega* **2021**, *6*, 4609.

[6] M. Ji, Q. Xia, H. Chen, Q. Cheng, Y. Liu, F. Li, *Environ. Process.* **2018**, *5*, 77.
